# The London Hospitals

**Published:** 1902-06-14

**Authors:** 


"Lord Mayor."

### THE LONDON HOSPITALS.

### MESSAGES FROM GREAT PERSONAGES.

TO-MORROW (Sunday) June 15th, is Coronation Hospital Sunday, and more than ordinary interest is being taken this year in the collections. The clergy and ministers of all denominations are combining in a supreme effort to raise £100,000 for the hospitals through the churches, and Mr. George Herring has promised to give 25 per cent. of the total amount contributed through the congregations to the Fund.

To help the cause a few of the great men and women of the nation have been invited to testify to the claims of the London hospitals upon all residents in the King's dominions in the Coronation year. His Grace the Archbishop of Canterbury, who is suffering from a sharp attack of gout, writes through his chaplain on June 10th:—

"With all his heart he commends the work of supporting the hospitals to the people of London. His Grace trusts that everything possible may be done to help them in their work, the noblest work that man can do."

The Right Hon. A. J. Balfour writes under the same date:—

"I have already spoken on behalf of the London hospitals, and can do little more than repeat what I have already said. They provide the most powerful machinery at our disposal for the alleviation of suffering, and it is to them we must largely look to supply the medical training and the opportunities for research on which the future of medicine so greatly depends. They are of as much importance, therefore, to the rich as to the poor, to the future as to the present, and should be regarded as in the fullest sense objects of national concern."

The Right Hon. Joseph Chamberlain writes under date of June 7th:—

"I heartily wish you success in your effort of mercy, which at this time of general thanksgiving and rejoicing will specially appeal to the British public."

Those who will be absent from London on Sunday may send their contributions to the Bank of England, or direct to the Lord Mayor at the Mansion House, E.C., to the account of the Hospital Sunday Fund.

